# MeImmS: Predict Clinical Benefit of Anti-PD-1/PD-L1 Treatments Based on DNA Methylation in Non-small Cell Lung Cancer

**DOI:** 10.3389/fgene.2021.676449

**Published:** 2021-05-20

**Authors:** Shipeng Shang, Xin Li, Yue Gao, Shuang Guo, Dailin Sun, Hanxiao Zhou, Yue Sun, Peng Wang, Hui Zhi, Jing Bai, Shangwei Ning, Xia Li

**Affiliations:** College of Bioinformatics Science and Technology, Harbin Medical University, Harbin, China

**Keywords:** DNA methylation, immunotherapy, non-small cell lung cancer, machine learning, immune regulatory genes

## Abstract

Immunotherapy has become an effective therapy for cancer treatment. However, the development of biomarkers to predict immunotherapy response still remains a challenge. We have developed the DNA Methylation Immune Score, named “MeImmS,” which can predict clinical benefits of non-small cell lung cancer (NSCLC) patients based on DNA methylation of 8 CpG sites. The 8 CpG sites regulate the expression of immune-related genes and MeImmS was related to immune-associated pathways, exhausted T cell markers and immune cells. Copy-number loss in 1p36.33 may affect the response of cancer patients to immunotherapy. In addition, SAA1, CXCL10, CCR5, CCL19, CXCL11, CXCL13, and CCL5 were found to be key immune regulatory genes in immunotherapy. Together, MeImmS discovered the heterogeneous of NSCLC patients and guided the immunotherapy of cancer patients in the future.

## Introduction

Non-small cell lung cancer (NSCLC) is the most common cancer type of lung cancer and one of the leading causes of cancer-associated deaths (Bray et al., 2018). Immunotherapy is an emerging cancer treatment and has provided significant clinical benefits to NSCLC. Immunotherapy includes adoptive T-cell therapy and immune checkpoint blockade (Kennedy and Salama, 2020). Programmed cell-death protein 1 ligand 1 (PD-L1) released by tumor cells bind to the programmed cell-death protein 1 (PD-1) present on cytotoxic T cells, which cause T cell exhaustion (Kuzume et al., 2020). Checkpoint inhibitors targeting CTLA-4, PD-1, and PD-L1 have yielded response in NSCLC and melanoma. A variety of monoclonal antibodies against PD-1/PD-L1 have been produced, such as nivolumab, pembrolizumab (Borghaei et al., 2015; Ribas et al., 2015). These PD-1/PD-L1 inhibitors can lead to stable regression of tumor cells. However, immunotherapy response is not universal, and only some patients can benefit from immunotherapy. Precise biological markers are essential for personalized immunotherapy. Tumor mutation burden, cytolytic activity (CYT), major histocompatibility complex (MHC) class I and the number of tumor-infiltrating lymphocytes can predict the response to immunotherapy (Zaravinos et al., 2019; Goodman et al., 2020). However, there is very little research on the role of DNA methylation in immunotherapy response.

Abnormal DNA methylation occurs in the early stage of carcinogenesis and presents different patterns in NSCLC. DNA methylation was an accurate biomarker for the prognosis and chemotherapy drug of NSCLC (Sandoval et al., 2013; Grasse et al., 2018). Recent research found that utilization of epigenetic targets is becoming an effective method of cancer treatment (Yoo and Jones, 2006). T cell exhaustion state is associated with DNA methylation, and inhibiting DNA methylation of activated CD8 T cells can maintain the function of T cells (Emran et al., 2019). Therefore, DNA methylation may be an effective predictor of the clinical benefit of immunotherapy.

In this study, we constructed DNA methylation immune Score (MeImmS), which can predict clinical benefit of anti-PD-1/PD-L1 immunotherapy in NSCLC patients based on DNA methylation. Multiple key immune regulatory genes were identified based on MeImmS, some of which have been confirmed in recent research to be closely related to the immune response.

## Materials and Methods

### Publicly Available Cohort Datasets and Preprocessing

Raw data of 78 NSCLC patients treated with anti-PD-1/PD-L1 therapy were obtained in the form of IDAT files from GEO database (GSE119144 and GSE126045). The files were parsed into R using the “ChAMP” and were normalized using BMIQ (Teschendorff et al., 2013; Tian et al., 2017). The beta value of each probe is calculated for subsequent analysis. The formula for DNA methylation beta value is as follows,

beta=signalB/(signalA+signalB+100)

which signal A represents unmethylated signal intensity and signal B represents methylated signal intensity.

In addition, patients were classified as responders if they showed partial response or stable disease for > 6 months, and patients who showed progressive disease (PD) or stable disease (SD) for < 6 months were classified as non-responders, including 20 responders and 58 non-responders. RNA-seq data of these NSCLC patients were downloaded from GSE135222 and GSE126044, including 11 responders and 23 non-responders. TPM of gene was calculated and normalized by Combat using R package “sva” (Leek et al., 2012).

Lung adenocarcinoma (LUAD) and lung squamous cell carcinoma (LUSC) datasets were downloaded from The Cancer Genome Atlas (TCGA), including DNA methylation, gene expression and somatic mutation. The DNA copy number (SNP 6.0) of LUAD and LUSC were collected from FireBrowse^1^.

### Construction of DNA Methylation Immune Score (MeImmS)

We developed a MeImmS pipeline that can evaluate the immunotherapy response in NSCLC. Firstly, differentially methylated CpG sites were identified by *t*-test at *p*-value threshold of 0.001 and methylation difference threshold of 0.15 between the responders and non-responders. Then, Least Absolute Shrinkage and Selection Operator (LASSO) regression model, RandomForest model and SVM model were constructed for predicting immunotherapy response of NSCLC patients based on DNA methylation of differentially methylated CpG sites using the R package “glmnet,” “randomForest,” and “e1071,” respectively. LASSO regression model was found to have the best predictive performance for immunotherapy response in these three models and identified 8 key CpG sites. Therefore, DNA Methylation Immune Score (MeImmS) was calculated by DNA methylation and weight of 8 CpG sites identified by LASSO regression model:

$$\text{MeImmS}=\sum_{\text{i}=1}^{\text{n}}\beta_{\text{i}}{\text{m}}_{i}+\text{c}$$

where m_i_ represents the DNA methylation of ith CpG site, β_i_ represents the weight of ith CpG site and c = −1.294. And MeImmS could divide NSCLC patients in responders and non-responders with a threshold of 0.

### MHC Score and CYT Score

The mean expression level of HLA-A, HLA-B, HLA-C, TAP1, TAP2, NLRC5, PSMB9, PSMB8, and B2M was calculated as MHC score (Lauss et al., 2017). CYT score was calculated based on geometric mean of granzyme A (GZMA) expression and perforin (PRF1) expression.

### Enrichment Score of Immune-Associated Pathway

We collected 1,793 genes of 17 immune-related signaling pathways from the ImmPort database^2^, and calculated the enrichment score of each pathway based on the gene expression data of LUAD and LUSC by using “GSVA” packages. The 17 immune-related signaling pathways are “Antigen Processing and Presentation,” “Antimicrobials,” “B cell receptor (BCR) Signaling Pathway,” “Chemokines,” “Chemokine Receptors,” “Cytokines,” “Cytokine Receptors,” “Interferons,” “Interferon Receptor,” “Interleukins,” “Interleukins Receptor,” “Natural Killer Cell Cytotoxicity,” “T cell receptor (TCR) signaling Pathway,” “TGFb Family Member,” “TGFb Family Member Receptor,” “TNF Family Members,” and “TNF Family Members Receptors.”

### Immune Cell Analysis

The proportion of immune cells was estimated based on gene expression data of LUAD and LUSC by using CIBERSORTx^3^. CIBERSORTx can use a deconvolution algorithm to compute the abundance of 22 cell types (Newman et al., 2019). Samples with *p* < 0.05 were selected for analysis because the proportion of immune cells of these samples was estimated more accurately.

### Statistical Analysis

The *T*-test and Wilcoxon rank sum test were used for comparisons of the proportion of immune cells and expression of exhausted T cell markers between MeImmS-High and MeImmS-Low, respectively. Mutation frequency of each gene was calculated by using R package “maftools” based on Mutation Annotation Format (MAF) of somatic mutation. Copy number variation region was identified by GISTIC 2.0. Five types of discretized copy number calls (-2, -1, 0, 1, and 2) for copy number variation region were determined. Logistic regression algorithm to calculate the copy number variation difference between the MeImmS-High and the MeImmS-Low. The R package “limma” was used to screen differential expression genes (adjusted *P* < 0.05 and fold-change > 1.5 or fold-change < 0.67) between MeImmS-High and MeImmS-Low. The results were considered statistically significant when the *p* < 0.05 in R versions 3.6.3.

## Results

### Identified of Immune-Associated CpG Sites

**FIGURE 1:**
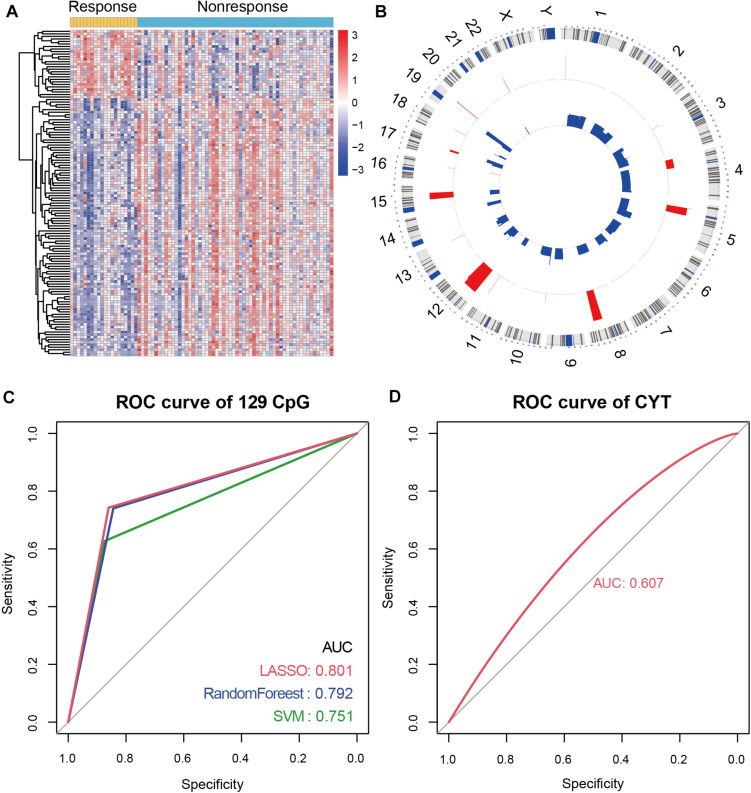
Immune-associated CpG sites in NSCLC. **(A)** Differential methylated CpG sites between responders and non-responders. Yellow represents responders and light blue represents non-responders. **(B)** Circos diagram of the distribution of differential methylation CpG sites on chromosomes. **(C)** In NSCLC patients with anti-PD-1/PD-L1 therapy, receiver operating characteristics (ROC) analysis of 129 differential methylation CpG sites in LASSO regression model, RandomForest model, and SVM model. The AUC is labeled. **(D)** ROC analysis of CYT score in NSCLC patients with anti-PD-1/PD-L1 therapy.

To characterize the differential DNA methylation pattern in NSCLC patients, we integrated the DNA methylation data of **78** NSCLC patients before receiving anti-PD-1/PD-L1 therapy. Of these patients, 20 patients were responders and 58 patients were non-responders. Next, 129 differential methylation CpG sites were identified between responders and non-responders. Of the 129 CpG sites, 27 CpG sites showed hypermethylation, and 102 CpG sites showed hypomethylation in responders (Figure 1A). In order to explore the genome position of these differentially methylated CpG sites, we analyzed the distribution of 129 CpG sites on chromosome. Most of the CpG sites exhibiting hypomethylation in responders were distributed on chromosome 3 and 11, while the CpG sites exhibiting hypermethylation were mainly distributed on chromosome 5 and 12 (Figure 1B).

To verify the role of 129 CpG sites in predicting immunotherapy response, we constructed prediction models of immunotherapy response. Firstly, we constructed the dataset containing 78 samples and 129 CpG sites (named “MeImm” dataset). We utilized the MeImm dataset to construct prediction models of response to immunotherapy by three machine learning methods, which were LASSO regression model, RandomForest model and support vector machine (SVM) model. The area under the curve (AUC) was used to compare the predictive power of the three models for immunotherapy response, when 10-fold cross-validation was performed. After 100 times sampling, it is found that the LASSO regression model has the best predictive performance. AUC of LASSO regression model, RandomForest model and SVM model were 0.801, 0.792, and 0.751 (Figure 1C).

CYT score is a useful tool to assess clinical benefit of immunotherapy. We compared the performance of CYT and DNA methylation in predicting response to immunotherapy. However, AUC of CYT score was only 0.607 in NSCLC patients receiving anti-PD-1/PD-L1 therapy, which was lower than predictive performance of 129 CpG sites for immunotherapy response (Figure 1D).

### Construction of DNA Methylation Immune Score

**FIGURE 2:**
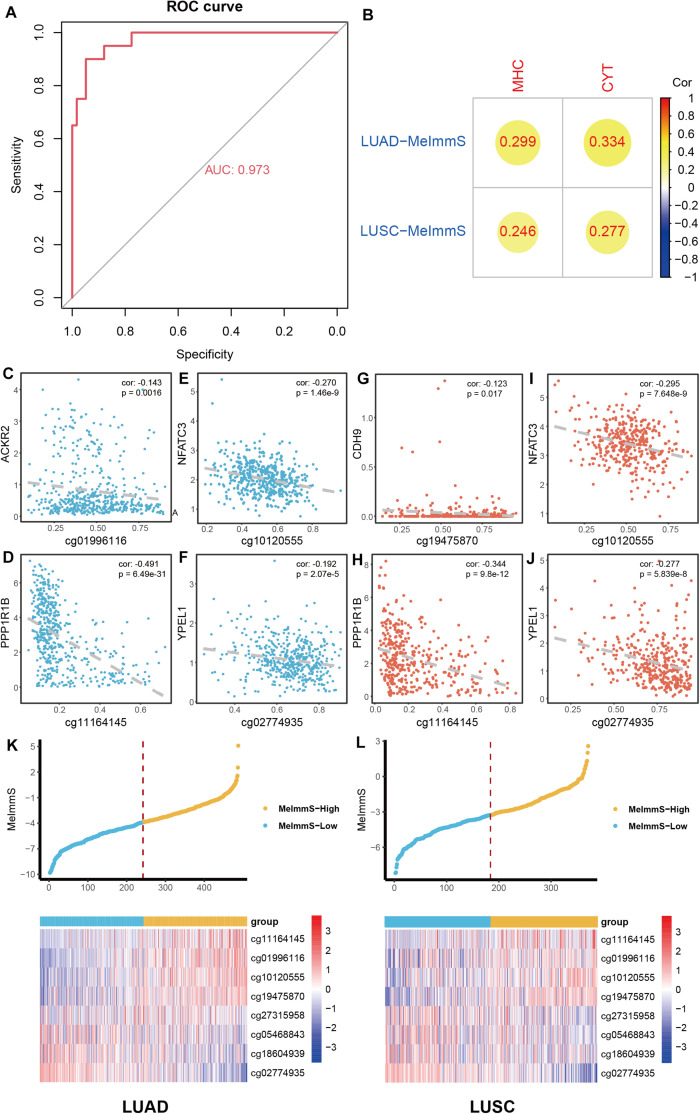
Construction of DNA methylation immune score. **(A)** In NSCLC patients with anti-PD-1/PD-L1 therapy, ROC analysis of the predictive ability of MeImmS on response to immunotherapy. **(B)** Correlation analysis of between MeImmS and MHC score, CYT score in LUAD and LUSC from TCGA database. The gradient from red to blue represents the degree of correlation between MeImmS and MHC or CYT. **(C–J)** Correlation analysis of between DNA methylation of immune-associated CpG sites and expression of immune-associated gene in NSCLC from TCGA database. Blue blots represent LUAD samples and orange blots represent LUAD samples. **(K,L)** Scatter diagram of MeImmS of and heatmap of 8 CpG sites in LUAD and LUSC. Yellow blots represent samples of MeImmS-High and light blue blots represent samples of MeImmS-Low.

To describe the immunotherapy response of each NSCLC patient, LASSO regression model was used to screen key CpG sites based on MeImm dataset. 8 CpG sites of 129 CpG sites were identified to construct DNA methylation immune score (MeImmS) with minimized lambda (lambda = 0.01060691). Next, MeImmS was constructed based on DNA methylation and weight of 8 CpG sites. In the MeImm dataset, the AUC of MeImmS was 0.973 in predicting response to immunotherapy (Figure 2A). The performance of MeImmS is significantly better than CYT in predicting immunotherapy response of NSCLC patients (Figure 1D).

To further verify the performance of MeImmS, we downloaded 486 LUAD samples and 370 LUSC samples from the TCGA database. Next, we calculated the MeImmS of patients in lung adenocarcinoma (LUAD) and lung squamous cell carcinoma (LUSC) from TCGA and found that MeImmS was significantly correlated with MHC score and CYT score (Figure 2B). cg01996116, cg1012055, cg1116414, cg02774935, and cg19475870 located in the promoter region of ACKR2, NFATC3, PPP1R1B, YPEL1, and CDH9, respectively. The DNA methylation level of these CpG sites was significantly negatively correlated with gene expression (Figures 2C–J). The deficiency of ACKR2 can increase the recruitment of natural killer cells and increase the lethality of tumors (Hansell et al., 2018). DNA methylation level of these CpG sites could inhibit gene expression and affect immune-related function. Cadherin 9 (CDH9) is an important protein that strengthens the interaction between immune lymphocytes and tumor cells (Durgeau et al., 2018; Mami-Chouaib et al., 2018). NFATC3, PPP1R1B, and YPEL1 play an important role in immune and carcinogenic pathways (Rao et al., 1997; Kelley et al., 2010; Klebanoff et al., 2017).

Finally, we divided the tumor samples of LUAD and LUSC into high DNA methylation immune score group (MeImmS-High) and low DNA methylation immune score group (MeImmS-Low) based on the median value of the DNA methylation immune score (Figures 2K,L).

### Correlation Analysis Between DNA Methylation Immune Score and Immune Markers

**FIGURE 3:**
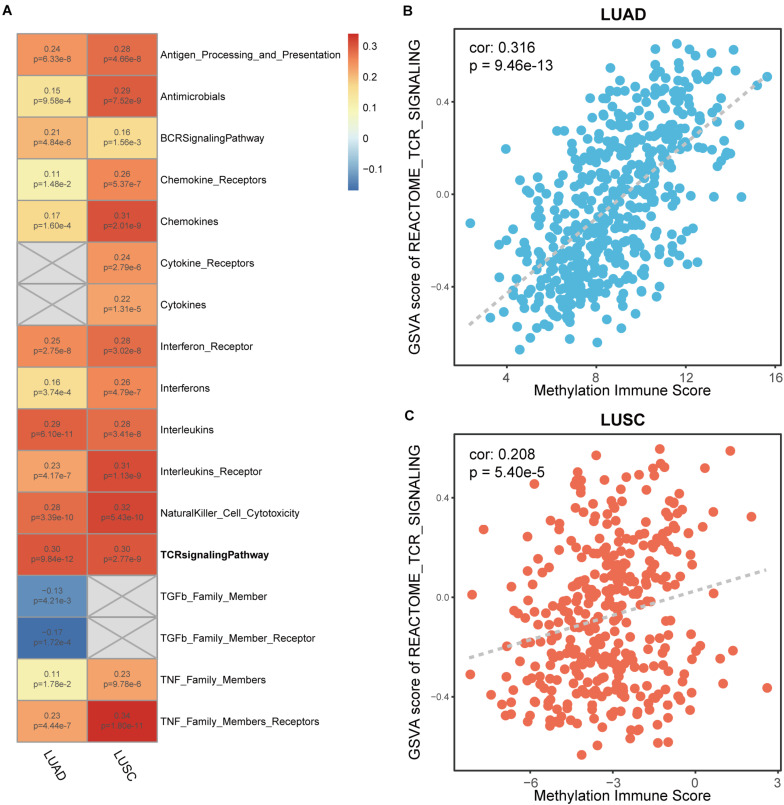
Correlation analysis of between enrichment score of immune-associated pathways and MeImmS in LUAD and LUSC. **(A)** Heat map of between GSVA score of 17 immune-associated pathways and MeImmS in LUAD and LUSC. Red represents a significant positive correlation between GSVA score of immune-associated pathway and MeImmS, and blue represents a significant negative correlation between GSVA score of immune-associated pathway and MeImmS. Gray represents that there is no correlation between the immune pathway and MeImmS. The numbers in the graph represent the correlation coefficient and *p*-value between GSVA score of immune pathway and MeImmS. **(B,C)** Scatter plot of between enrichment score of TCR signaling pathway and MeImmS in LUAD and LUSC.

To determine MeImmS is associated with immune function, immune-associated pathway enrichment score of each sample was calculated by using GSVA in LUAD and LUSC, respectively. In 17 immune-associated pathways, MeImmS was significantly positively associated with enrichment score of 13 immune-associated pathways in LUAD and LUSC (Figure 3A). In immunotherapy, tumor cells are destroyed by activating the function of T cells. Therefore, the T cell receptor (TCR) signaling pathway plays an important role in immunotherapy. We found that MeImmS was strongly correlated with T cell receptor signaling pathways. Moreover, MeImmS was also strongly correlated with enrichment score of T cell receptor signaling pathway from REACTOME in LUAD and LUSC (Figures 3B,C).

**FIGURE 4:**
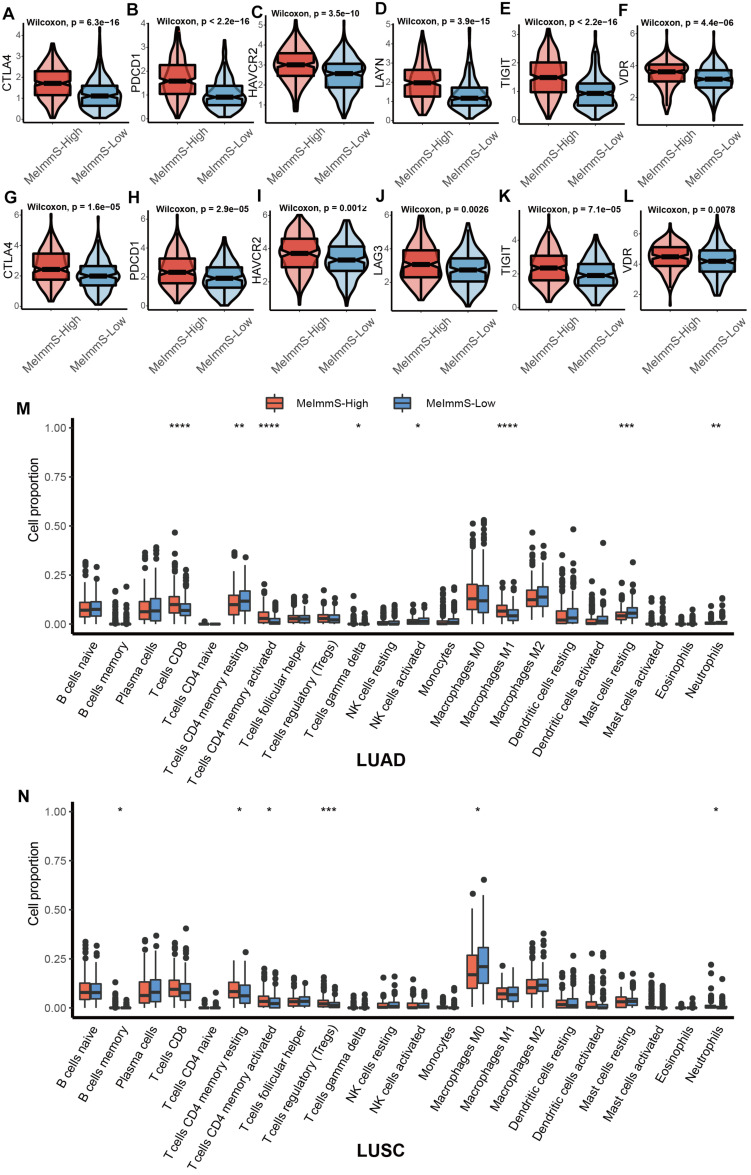
Comparison of immune related markers between MeImmS-High and MeImmS-Low. **(A–L)** Violin plots show the expression of exhausted CD8+ T cell markers between MeImmS-High and MeImmS-Low. **(M,N)** Boxplot shows the immune cell proportions between MeImmS-High and MeImmS-Low in LUAD **(M)** and LUSC **(N)**.

Studies have found that exhausted T cell markers can measure the patients’ response to immunotherapy and patients with more exhausted CD8+ T cells are more suitable for immunotherapy (Snell et al., 2018). We collected 7 exhausted T cell markers: CTLA-4, HAVCR2, LAYN, LAG3, PDCD1, TIGIT, and VDR. CTLA-4, HAVCR2, PDCD1, TIGIT and VDR were significantly different between MeImmS-High and MeImmS-Low, and both show high expression in MeImmS-High (Figures 4A–L).

In addition, by comparing the difference between the MeImmS-High and MeImmS-Low in the proportion of immune cells, it was found that the proportion of activated CD4 memory T cells was significantly higher in the MeImmS-High (Figures 4M,N). Studies have found that patients with functional CD4 immunity and high expression of PD-L1 exhibited response rates of 70%, revealing the important role of CD4 immunity in anti-PD-L1/PD-1 therapy (Zuazo et al., 2019). The MeImmS was significantly associated with immune pathways and immune markers, suggesting that the MeImmS can be used to predict the response to immunotherapy in NSCLC patients.

### Genomic Mutation Between MeImmS-High and MeImmS-Low

**FIGURE 5:**
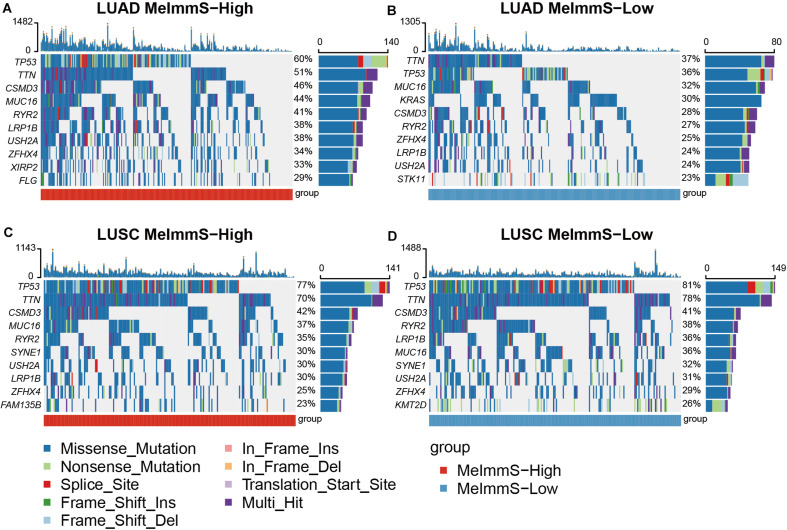
Comparison of gene mutation frequency between MeImmS-High and MeImmS-Low. **(A,B)** Gene mutation frequency of MeImmS-High and MeImmS-Low in LUAD. **(C,D)** Gene mutation frequency of MeImmS-High and MeImmS-Low in LUSC.

We calculated the gene mutation frequency in LUAD and LUSC using somatic mutation data, respectively. In LUAD and LUSC, we found that six genes with the highest mutation frequency were the same, which were TP53, TTN, MUC16, CSMD3, RYR2, and LRP1B (Figure 5). In addition, we found that TP53, TTN, and CSMD3 mutation frequencies were significantly different between MeImmS-High and MeImmS-Low in LUAD (Chi-square test TP53: *p* = 8.96e-4, TTN: *p* = 0.014, CSMD3: *p* = 0.0095). It suggests that TP53 mutation can guide the immunotherapy effect of LUAD patients. In previous studies, TP53 mutation can be used as a predictor of anti-PD-1/PD-L1 immunotherapy in LUAD patients (Dong et al., 2017). CSMD3 is a transmembrane protein, which plays a significant role in protein-protein interaction and immune response (Ahn et al., 2014). Therefore, mutation of TP53, TTN, and CSMD3 maybe have the potential biomarker to predict the immunotherapy response of LUAD patients.

The study found that the oncogene KRAS is the main driving factor of LUAD, so we further analyzed the KRAS in LUAD. It was found that among the 65 patients with KRAS mutation in the MeImmS-Low of LUAD, nearly half of them had KRAS c.34G > T (p.Gly12Cys) (31/65) mutation. The second is KRAS c.35G > T (p.Gly12Val) mutation (12/65). However, in the 61 patients with KRAS mutation in the MeImmS-High of LUAD, the highest mutation frequency site of KRAS is c.35G > T (p.Gly12Val), the mutation frequency was 17/61, and the KRAS c.34G > T (p.Gly12Cys) site mutation frequency is only 16/61. It indicates that LUAD patients with KRAS c.34G > T (p.Gly12Cys) mutation may have poor immunotherapy effect and are not suitable for immunotherapy.

Next, we analyzed the copy number variation difference between MeImmS-High and MeImmS-Low in LUAD and LUSC, respectively. We identified 411 copy number variation regions in LUAD, of which 228 regions were copy number amplification and 183 regions were copy number deletion. And 347 copy number variation regions were identified in LUSC, of which 181 regions were copy number amplification and 166 regions were copy number deletion. Finally, 80 immune-associated copy number variation regions were identified in LUAD and 71 copy number variation regions were identified in LUSC using logistic regression algorithm (*p* < 0.05).

### Key Regulatory Gene of Immunotherapy

**FIGURE 6:**
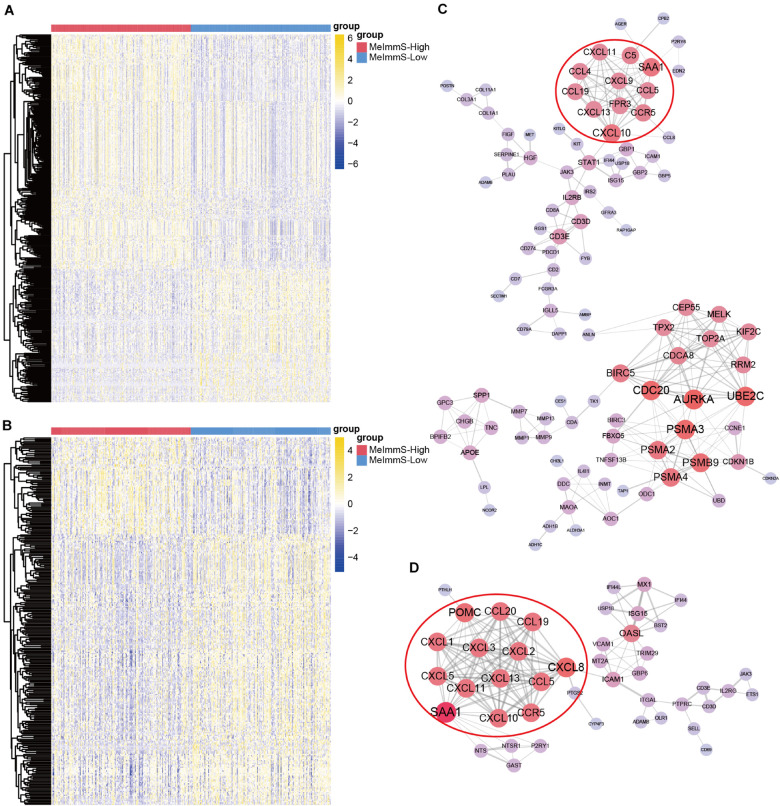
Identification of key regulatory gene of immunotherapy. **(A,B)** Differentially expressed genes between MeImmS-High and MeImmS-Low in LUAD **(A)** and LUSC **(B)**. **(C,D)** Protein-protein interaction network of differentially expressed genes in LUAD **(C)** and LUSC **(D)**.

We identified 381 and 288 differently expressed genes between MeImmS-High and MeImmS-Low in LUAD and LUSC, respectively (Figures 6A,B). Then, enrichment analysis of differently expressed genes was performed in LUAD and LUSC. In the LUAD, we found that the differently expressed genes between MeImmS-High and MeImmS-Low were significantly enriched in immune-related biological function and signaling pathway, such as leukocyte migration, antigen binding and cytokine activity, immunoglobulin complex and IL-17 signaling pathway (Supplementary Figures 1A–D). In LUSC, we also found that the differently expressed genes between the two immune subgroups were significantly enriched in immune-related biological function and signaling pathway, such as lymphocyte migration, chemokine activity, and chemokine signaling pathway (Supplementary Figures 1E–H). In summary, the differentially expressed genes between MeImmS-High and MeImmS-Low in LUAD and LUSC were mainly enriched in immune-related function and pathway. It suggests that MeImmS-High and MeImmS-Low divided by MeImmS have significant differences in immune function, which further proved that MeImmS can be used as an indicator to evaluate the immunotherapy response of NSCLC.

In order to further explore the key regulatory factors affecting immunotherapy response, we calculated the protein-protein interaction relationship between differentially expressed genes by using String database^4^ in LUAD and LUSC, respectively. Finally, we got 303 relationship pairs in LUAD, and 202 relationship pairs in LUSC (Figures 6C,D). We found that the protein-protein interaction network of LUAD, the key immune checkpoint PD-1 and PD-L1 are all related to CD3D. Interestingly, researchers found that CD3D is the T cell-related marker, and CD3D defects can cause severe immunodeficiency (Rowe et al., 2018), which indicates the potential role of CD3D in predicting the immunotherapy response. It further shows that CD3D can be used as a key regulatory factor of immunotherapy response for cancer patients.

Next, we screened key regulatory genes in LUAD and LUSC, respectively. In the protein-protein interaction network of LUAD, there are 26 key regulatory genes with degree >10. In the protein-protein interaction network of LUSC, there are 15 key regulatory genes with degree >10. Interestingly, SAA1, CXCL10, CCR5, CCL19, CXCL11, CXCL13, and CCL5 had significant regulatory effect in LUAD and LUSC. Moreover, CXCL10, CXCL11, and CXCL13 all belong to the CXC chemokines subfamily. In addition, studies have found that the highly expressed CXCL10 is an important marker of immune response (Antonelli et al., 2014), so the CXC subfamily genes may have regulatory effect on immunotherapy.

In order to further verify the immunomodulatory effects of these genes in cancer patients, we downloaded RNA-seq data of 348 urothelial carcinoma patients (IMvigor210) who received anti-PD-L1 (Atezolizumab) treatment (Necchi et al., 2017). Then we identified 1,654 genes related to immunotherapy response. Interestingly, we found that SAA1, CXCL10, CCL5, and CXCL13 also had significant differences between responders and non-responders in IMvigor210 dataset. It suggests that SAA1, CXCL10, CCR5, CCL19, CXCL11, CXCL13, and CCL5 have important function in immunotherapy for cancer patients, and may be used as key regulator genes of immunotherapy.

## Discussion

DNA methylation is an important epigenetic modification that can regulate gene expression without changing the DNA sequence. DNA methylation plays an important regulatory role in the differentiation of immune cells, which may have an impact on the response to immunotherapy (Jones et al., 2019). Previous research has found that DNA methylation of CTLA-4 could predict response of anti-CTLA-4 and anti-PD-1 immune checkpoint blockage in melanoma (Goltz et al., 2018). DNA methylation of costimulatory/immune checkpoint molecules could reflect tumor immunogenicity of pan-cancer (Berglund et al., 2020). Hence, DNA methylation may be an important predictor of immunotherapy response.

Lung tumor is a heterogeneous disease, which has different response to anti-PD-1/anti-PD-L1 treatments. In this study, MeImmS was constructed based on 8 CpG sites by using machine learning, which could predict the immunotherapy response of NSCLC patients. DNA methylation of the 8 CpG sites affected the expression level of multiple immune genes, which may have an important impact on the immune response. In addition, MeImmS was also significantly associated with predictive markers of immunotherapy, such as MHC score and cytolytic activity. Excellent predictive performance of MeImmS implied the predictive ability of DNA methylation in immunotherapy response. With increasing DNA methylation data for immunotherapy, MeImmS pipeline for predicting immunotherapy response based on DNA methylation can be extended to more cancers.

Besides, genomic variation is also an important factor affecting the response of immunotherapy. Identifying the genomic variant of cancer patient may be helpful for improving the treatment. Seven key immune regulatory factors were identified in NSCLC samples, which could play an important role in immunotherapy. In addition, CXCL10 and CXCL11 were closely related to CD8^+^ tumor-infiltrating lymphocytes, which may predict benefit from PD-1 blockade therapy (Wu et al., 2019).

Our research shows that DNA methylation plays an important role in regulating and predicting immunotherapy response. And DNMT inhibitors have been approved for the treatment of myelodysplastic syndrome and acute myeloid leukemia (Zhou et al., 2021). Therefore, the combined application of epigenetic inhibitors and immune checkpoint inhibitors may be an effective therapy for cancer treatment in the future.

## Data Availability Statement

The original contributions presented in the study are included in the article/Supplementary Material, further inquiries can be directed to the corresponding author/s.

## Author Contributions

XiaL, SN, and JB designed this work. SS performed statistical analysis and bioinformatics analysis, and wrote the manuscript. XinL and YG performed the bioinformatics analysis. SG, DS, HZ, and YS performed statistical analysis. PW and HZ revised the manuscript. All authors contributed to the article and approved the submitted version.

## Conflict of Interest

The authors declare that the research was conducted in the absence of any commercial or financial relationships that could be construed as a potential conflict of interest.

## Footnotes

1 http://firebrowse.org/

2 https://www.immport.org/

3 https://cibersortx.stanford.edu/

4 https://string-db.org/
