# Supplementary figures and images for: MeImmS: Predict Clinical Benefit of Anti-PD-1/PD-L1 Treatments Based on DNA Methylation in Non-small Cell Lung Cancer
Source: Front Genet. 2021 May 20;12:676449. doi: 10.3389/fgene.2021.676449 (PMC8173132; doi:10.3389/fgene.2021.676449)

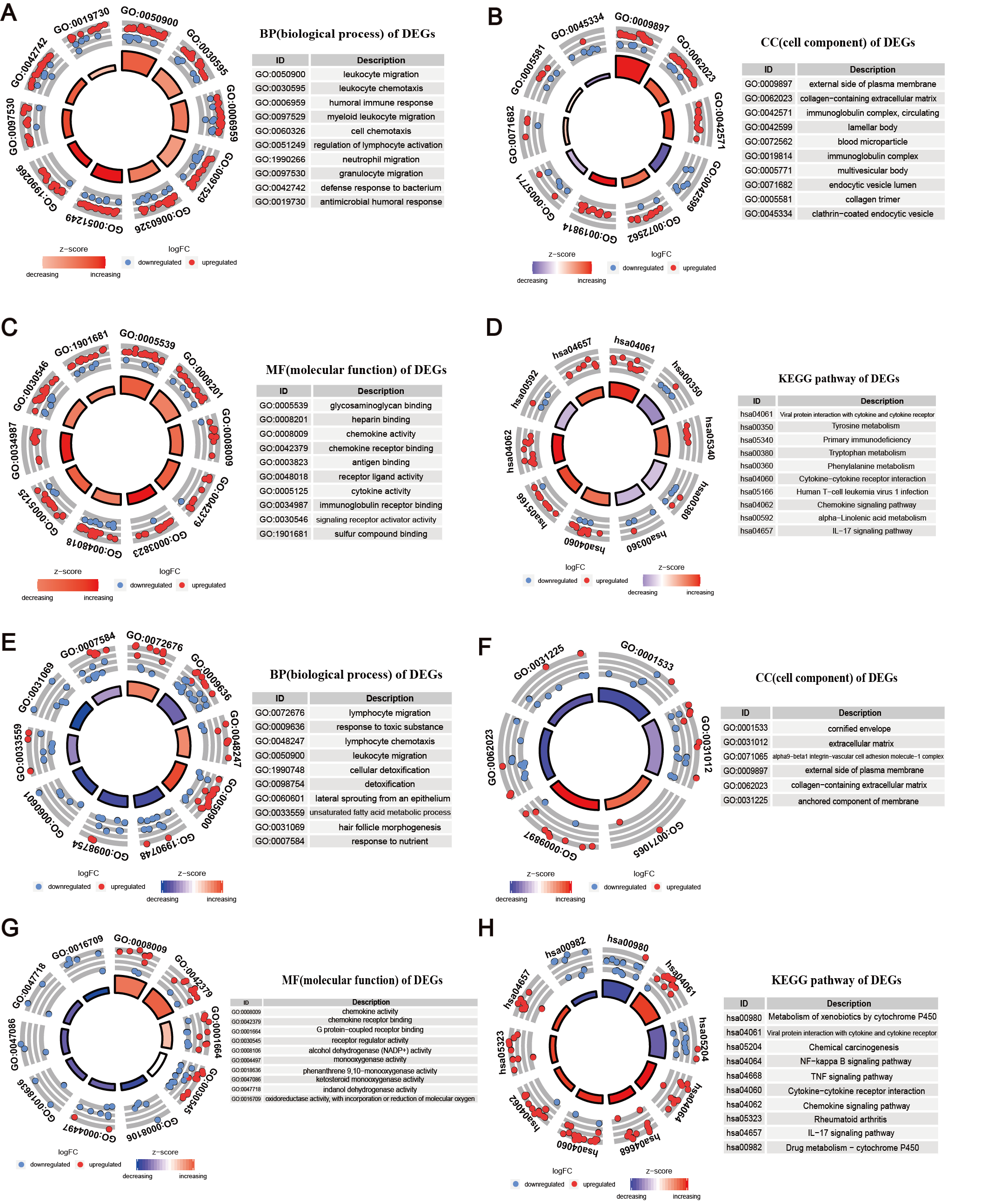

Supplement: Supplementary file 1 [file Image_1.TIF]
