# Supplementary material for: MeImmS: Predict Clinical Benefit of Anti-PD-1/PD-L1 Treatments Based on DNA Methylation in Non-small Cell Lung Cancer
Source: Front Genet. 2021 May 20;12:676449. doi: 10.3389/fgene.2021.676449 (PMC8173132; doi:10.3389/fgene.2021.676449)
Supplement: Supplementary file 2 [file Data_Sheet_1.docx]

***Supplementary Material***

**Supplementary Figures**

**Supplementary Figure 1.** Functional Enrichment Analysis of differentially expressed genes between MeImmS-High and MeImmS-Low in LUAD and LUSC. (A-D) In LUAD, functional Enrichment Analysis of genes on biological process (A), cell component (B), molecular function (C) and KEGG pathway (D). (E-H) In LUSC, functional Enrichment Analysis of genes on biological process (E), cell component (F), molecular function (G) and KEGG pathway (H).

**Supplementary Tables**

**Supplementary Table 1. Weight of 8 CpG sites.** This file contains 8 CpG sites and their weights required to calculate MeImmS.
